# Eosinophilic gastritis without peripheral eosinophilia and atopy: A case report

**DOI:** 10.1002/ccr3.6005

**Published:** 2022-06-27

**Authors:** Aakash Mishra, Tulsi Ram Bhattarai, Aman Mishra, Shekhar Poudel

**Affiliations:** ^1^ Kathmandu Medical College Teaching Hospital Kathmandu Nepal; ^2^ Department of Internal Medicine Kathmandu Medical College Teaching Hospital Kathmandu Nepal; ^3^ Maharajgunj Medical Campus Institute of Medicine Kathmandu Nepal; ^4^ Department of Gastroenterology Kathmandu Medical College Teaching Hospital Kathmandu Nepal

**Keywords:** antral ulcer, eosinophilia, eosinophilic gastritis, eosinophilic gastrointestinal disease, gastroenteritis

## Abstract

Eosinophilic gastritis (EG) is characterized by eosinophilic infiltration of any gastric layers. We report a 65‐year‐female presenting with abdominal pain and vomiting for two months. Chronic gastritis not responding to empirical treatment interrogated further investigations. In the absence of atopy and peripheral eosinophilia, successful treatment of a large solitary antral ulcer with steroids upheld the diagnosis of EG.

## 1 INTRODUCTION

The eosinophilic gastrointestinal disorders (EGID) can affect any segment of the gastrointestinal tract and based on the anatomical site involved, it is classified into five types, namely, eosinophilic esophagitis, eosinophilic gastritis (EG), eosinophilic enteritis, eosinophilic gastroenteritis, and eosinophilic colitis of which EG is the least common.^1^ EG is a rare inflammatory condition characterized by eosinophilic infiltration involving any layer of the stomach wall and the absence of an identifiable cause of eosinophilia. EG is more common among the elderly with female predominance.^2^ The risk factors and pathogenesis of EGID are largely unknown and the clinical manifestations are nonspecific. The diagnosis is challenging, requires a high index of clinical suspicion, and mandates endoscopy and biopsy for accurate diagnosis. Histopathological examination (HPE) with ≥30 eosinophils/high‐power field (HPF) in the absence of a known cause of eosinophilia is diagnostic.^3^, ^4^ Herein, we present a case of EG and aim to discuss its clinical manifestations, diagnostic modalities and challenges, and appropriate management.

## 2 CASE REPORT

A 65‐year‐female presented to the out‐patient clinic with complaints of persistent pain in the left upper quadrant and multiple episodes of vomiting for two months. The pain aggravated with the consumption of food, restricting her intake and she inevitably lost approximately 5 kg of body weight. The vomitus was nonblood stained, nonbilious, consisting of recently ingested food particles. There was no fever, night sweats and skin lesions noted. The past medical history was insignificant in particular to allergic diseases such as asthma, atopic dermatitis, allergic rhinitis, food intolerance, or known drug allergy. Family history was negative for the same. She is a nonsmoker and nonalcoholic.

She was initially evaluated in another center two months back with the same complaints where upper gastrointestinal endoscopy (UGIE) was performed and an ulcer in the antrum was noted. The specimen was not sent for HPE. She was prescribed clarithromycin‐based *Helicobacter pylori* regimen empirically. The symptoms did not resolve, worsened instead, and was prescribed with levofloxacin‐based *Helicobacter pylori* regimen.

On examination, the vital signs were stable. Conjunctival pallor and tenderness over the left upper quadrant were noted. There was no lymphadenopathy, hepatosplenomegaly, or ascites. The hemogram revealed a hemoglobin level of 9.1 gm% with normal total and differential leucocyte count. The absolute eosinophil count was 147/mm^3^. Fecal occult blood test was positive.

**FIGURE 1:**
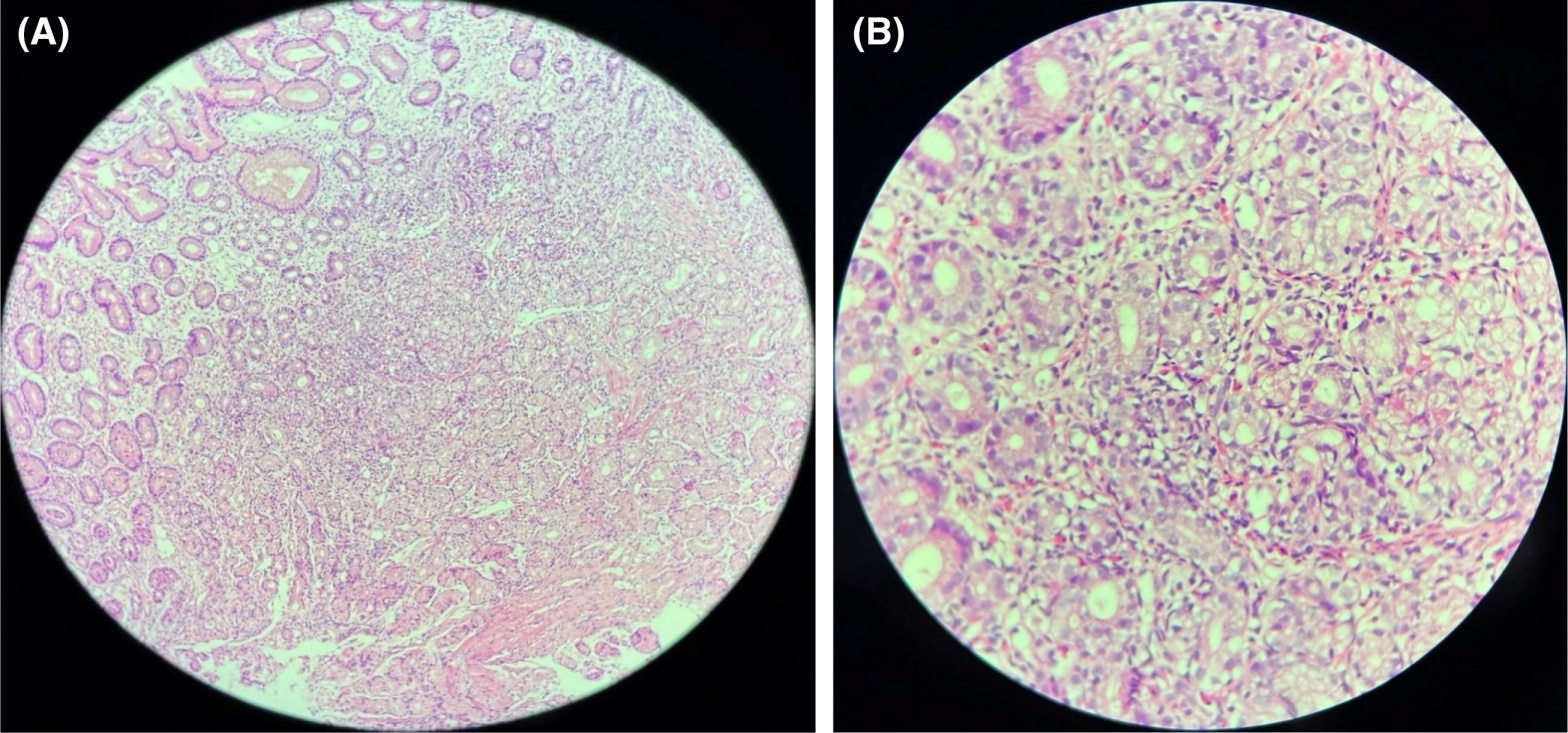
Illustrative gastric biopsy in eosinophilic gastritis. (A) Low‐power view of the antral mucosa showing diffuse eosinophilic infiltration in the lamina propria (hematoxylin and eosin stain; 100X magnification). (B) High‐power view of the lamina propria eosinophils (hematoxylin and eosin stain; 400X magnification)

The upper endoscopy examination was repeated at our institution, showing a large ulcer at the antrum along the lesser curvature. Multiple tissue biopsies were taken and sent for HPE. A computed tomography (CT) scan of the abdomen revealed focal and asymmetrical wall thickening measuring 6.5 mm along the lesser curvature of the stomach and extending to a length of 2.6 cm. No evidence of perigastric lymphadenopathy was seen. HPE showed chronic active ulcerative inflammation of antral mucosa, infiltration of lamina propria with eosinophils which constituted 54/HPF, lymphocytes, and plasma cells. No evidence of *Helicobacter pylori*‐like organisms was reported and HPE was suggestive of EG (Figure 1A,B).

Stool examinations were normal and showed no evidence of ova, cyst, or parasites. Urinalysis, liver, and renal functions were in the normal range. Serum IgE levels were normal. Colonoscopy, chest radiographs, and connective tissue disorder tests were unremarkable. She was treated with a proton pump inhibitor and prednisolone 40 mg daily for four weeks and was tapered over the next four weeks. Her symptoms improved gradually, and a UGIE performed after eight weeks showed complete remission of the ulcer.

## 3 DISCUSSION

Eosinophilic gastritis, also known as allergic gastritis, is a rare disease characterized by gastrointestinal symptoms, eosinophilic infiltration in the biopsy specimen, peripheral eosinophilia, and an absence of parasitic or extraintestinal disease.^5^ EG has been classified based on localization of eosinophil accumulation in different layers (mucosal type, muscular type, and subserosal type).^6^ Given the rarity of the diagnosis, the etiology, risk factors, prevalence of EG are largely unknown. Several studies have postulated allergic etiology and the presence of atopy in approximately 75% of all EGIDs.^7^

The clinical manifestations of EG are not specific. Our patient presented with complaints of abdominal pain, vomiting, and anemia. At presentation, our working diagnosis was chronic gastritis with a suspicion of malignancy based on age, chronicity, nonspecific symptomatology, and no‐response to prior treatment with clarithromycin and consecutive therapy with levofloxacin‐based *H. pylori* regimen. Further workup revealed a large antral ulcer with eosinophilic infiltration into the lamina propria (54/HPF), characteristic of the mucosal type of EG. Our patient had a hemoglobin level of 9.1 gm%, which can be attributed to the chronic blood loss from the ulcer and fecal occult blood test positivity. The total leucocyte count was 4900/mm^3^ and the differential count showed only 3% eosinophils with an absolute eosinophil count of 147/mm^3^. Solitary antral ulcer in the absence of atopy, known allergy, peripheral eosinophilia, normal serum IgE levels make our case rare. Misdiagnosis would warrant surgical treatment for chronic nonresolving gastritis and poor sequelae for the patient.

It is of utmost importance to rule out potential etiologies associated with eosinophilic infiltration of the gastric mucosa such as *H. pylori* or gastrointestinal helminth infections, hyper‐eosinophilic syndrome, malignancy, connective tissue disorders, drug hypersensitivity, or inflammatory bowel diseases. Detailed history, clinical examination, followed by relevant investigations were carried out to rule out the differentials, which yielded insignificant results.

The etiology and treatment is not well understood and hence primary treatment is symptomatic management. Based on the literature, the role of steroids and dietary modifications is effective.^8^, ^9^ Despite the lack of enough evidence for the role of steroids and dietary modifications, we used steroids, to which a dramatic response was observed, along the lines of existing literature and supporting our diagnosis of EG despite the absence of peripheral eosinophilia and atopy. Our finding parallels the existing literature on the use of steroids in the management of EG.

Diagnosis of EG, in the context of chronic gastritis or ulcers, can perplex malignancies and impose challenges to clinicians, especially when symptoms are unspecific. The laboratory parameters may be inconclusive, so endoscopic findings and HPE uphold the diagnosis. EG should be considered when gastrointestinal symptoms persist and do not respond to empirical treatment.

## AUTHOR CONTRIBUTION

Aakash Mishra (AM1) = Study concept, Data collection, Original draft preparation. Tulsi Ram Bhattarai (TRM) and Shekhar Poudel (SP) = Study concept, Treatment of the patient. Aman Mishra (AM2) = Histopathological studies and Editing of the manuscript. SP = Senior author and Manuscript reviewer. All authors were involved in reviewing the final draft of the manuscript. All authors have made a significant contribution to preparing the case report.

## CONFLICTS OF INTERESTS

Nothing to state.

## CONSENT

Written informed consent was obtained from the patient for publication of this case report and accompanying images. A copy of the written consent is available for review by the Editor‐in‐Chief of this journal on request.

## Data Availability

The data that support the findings of this study are available from the corresponding author upon reasonable request.
